# Screen, Notify, See, and Treat: Initial Results of Cervical Cancer Screening and Treatment in Rwanda

**DOI:** 10.1200/GO.20.00147

**Published:** 2021-04-30

**Authors:** Marie-Aimee Muhimpundu, Fidele Ngabo, Felix Sayinzoga, Jean Paul Balinda, John Rusine, Sardis Harward, Arielle Eagan, Sara Krivacsy, Alice Bayingana, Jean Claude Uwimbabazi, Jean Damascene Makuza, Jean de Dieu Ngirabega, Agnes Binagwaho

**Affiliations:** ^1^ Rwanda Biomedical Center, Kigali, Rwanda; ^2^ National Reference Laboratory, Kigali, Rwanda; ^3^ The Dartmouth Institute for Health Policy and Clinical Practice, Lebanon, NH; ^4^ University of Global Health Equity, Kigali, Rwanda; ^5^ Clinical Microbiology Laboratory, CHU de Liège, University of Liege, Liege, Belgium; ^6^ Harvard Medical School, Boston, MA; ^7^ Geisel School of Medicine, Dartmouth College, Hanover, NH

## Abstract

**PURPOSE:**

To describe the first year results of Rwanda's Screen, Notify, See, and Treat cervical cancer screening program, including challenges encountered and revisions made to improve service delivery.

**METHODS:**

Through public radio broadcasts, meetings of local leaders, church networks, and local women's groups, public awareness of cervical cancer screening opportunities was increased and community health workers were enlisted to recruit and inform eligible women of the locations and dates on which services would be available. Screening was performed using human papillomavirus (HPV) DNA testing technology, followed by visual inspection with acetic acid (VIA), and cryotherapy, biopsy, and surgical treatment for those who tested HPV-positive. These services were provided by five district hospitals and 15 health centers to HIV-negative women of age 35-45 and HIV-positive women of age 30-50. Service utilization data were collected from the program's initiation in September 2013 to October 2014.

**RESULTS:**

Of 7,520 cervical samples tested, 874 (11.6%) screened HPV-positive, leading 780 (89%) patients to undergo VIA. Cervical lesions were found in 204 patients (26.2%) during VIA; of these, 151 were treated with cryoablation and 15 were referred for biopsies. Eight patients underwent complete hysterectomy to treat advanced cervical cancer. Challenges to service delivery included recruitment of eligible patients, patient loss to follow-up, maintaining HIV status confidentiality, and efficient use of consumable resources.

**CONCLUSION:**

Providing cervical cancer screening services through public health facilities is a feasible and valuable component of comprehensive women's health care in resource-limited settings. Special caution is warranted in ensuring proper adherence to follow-up and maintaining patient confidentiality.

## INTRODUCTION

Cervical cancer is the leading cause of oncologic morbidity and mortality among women across sub-Saharan Africa, including Rwanda.^1^ Cervical cancer disproportionately affects low- and middle-income countries (LMICs): 85% of cases are estimated to occur in developing regions, with 17% of cases in sub-Saharan Africa alone.^2–4^ Because of large and growing disparities in access to preventive, screening, and curative services, LMICs are expected to bear 70% of global mortality because of cervical cancer by 2030.^5,6^ In Rwanda, cervical cancer accounts for 28.8% of all cancer diagnoses in women.^1^ Alarmingly, over the past 30 years, cervical cancer incidence appears to have increased in all regions of the world except for high-income countries.^4^

**CONTEXT**

**Key Objective**
What is the feasibility of a human papillomavirus testing screening program for the prevention of cervical cancer in Rwanda?
**Knowledge Generated**
Rwanda's Screen, Notify, See, and Treat program, conducted in 2013, was effective and feasible despite challenges in maintaining patient follow-up and achieving efficient use of consumable resources. Existing primary healthcare delivery structures and networks of community health workers in Rwanda were successfully leveraged in the screening program.
**Relevance**
Given recent changes in WHO recommendations, this program should be reinstated with revisions that address the challenges identified in order to reach global goals of cervical cancer elimination. A robust human papillomavirus screening program could be more appropriate in reducing the burden of cervical cancer than the existing widespread use of visual inspection with acetic acid.

In 2011, Rwanda made an important first step in reducing cervical cancer incidence and mortality by developing a successful, nationwide school-based human papillomavirus (HPV) vaccination program. In the first year of the program, 93.2% of girls in their sixth year of primary school were fully vaccinated for HPV with Gardasil, a quadrivalent HPV vaccine manufactured by Merck & Co (New York, NY).^7^ The program achieved further success in 2012 by reaching 96.6% of the target population after a catch-up period.^8^

In 2013-2014, baseline data on HPV prevalence were determined among a sample of 2,508 women of age 18-69 in Kigali.^9^ High-risk (HR) HPV strains were found in 22%,^9^ a higher prevalence compared with other countries with high rates of cervical cancer, such as India (12%)^10^ and Columbia (10%); this confirmed the epidemiological imperative for a comprehensive screening program in Rwanda.^11^ Several prevalence studies in Rwanda have confirmed that HR HPV prevalence is significantly higher among HIV-positive women and decreases less with age compared with HIV-negative women.^12–14^

Rwanda's comprehensive cervical cancer program expanded again in 2013, through a partnership with Qiagen (Hilden, Germany), which supported the introduction and set-up of cervical cancer screening facilities at five district hospitals and 15 health centers.^8^ However, the program was discontinued in the following year because of the cost of the test. In this article, the results from the first 12 months of Rwanda's cervical cancer screening program are presented along with discussion of implementation challenges and the potential for renewed scale-up.

## METHODS

### Program Preparation

In March 2013, the Ministry of Health (MoH) convened a Technical Working Group composed of representatives from the Maternal and Child Health, Non-Communicable Diseases, and HIV and STIs (sexually transmitted infections) Divisions, Health Communication Center, clinical services, and the National Reference Laboratory. Planning was headed by the MoH and the Rwanda Biomedical Center along with the WHO, Partners in Health, and the Centers for Disease Control and Prevention.^7–9^ In keeping with the values of decentralization and rights-based service delivery, this Working Group's first priority was to engage stakeholders from a variety of backgrounds for guidance and support for the program's development. MoH officials, Directors of District Hospitals, heads of health centers, civil society groups involved in cancer-related causes, and community leaders (particularly those representing women's groups) were invited to contribute guidance for the program during workshops and planning sessions. This process of consultation and collaborative program design ensured that insights from diverse perspectives were considered and that future stakeholders were invested in the program's success from its inception.

Following these discussions, a thorough situation analysis was conducted to determine the burden of cervical cancer disease in Rwanda and to inform decisions regarding the feasibility and scope of a comprehensive cervical cancer program. The Non-Communicable Disease Technical Working Group at MoH undertook a survey of existing services and resources that could be adapted for cervical cancer screening and determined whether required elements could reasonably be acquired or developed. Several pre-existing components of the Rwandan health system were well-positioned to accommodate cervical cancer screening: the nation's robust network of approximately 45,000 community health workers (CHWs) could be used to recruit eligible women and ensure appropriate follow-up, and well-established primary healthcare delivery strategies could be expanded to make screening available at the local level.

### Screening Strategy

**FIG 1:**
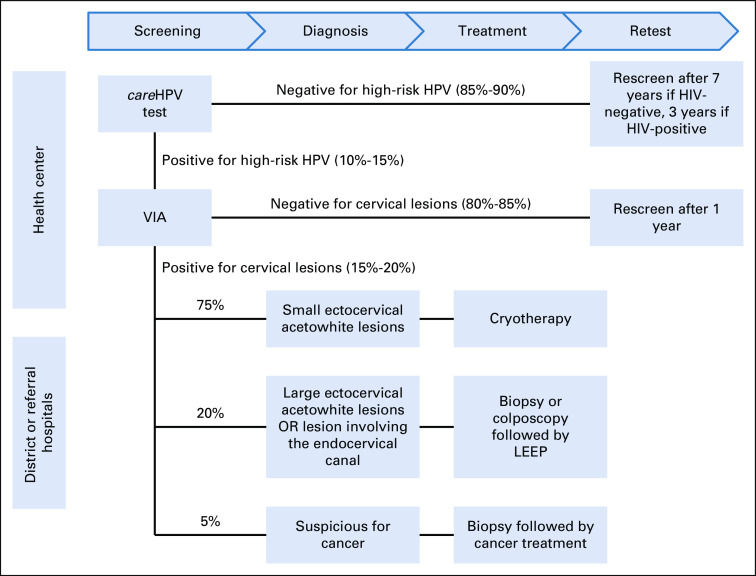
Algorithm for cervical cancer screening and follow-up services. HPV, human papillomavirus; LEEP, loop electrosurgical excision procedure; VIA, visual inspection with acetic acid.

To provide appropriate care to all women, a step-wise screening and treatment strategy that combines testing for HPV DNA, visual inspection with acetic acid (VIA), cryotherapy, biopsy, and surgical treatment was adopted.^8^ Screening services were offered to all women of age 35-45 and to HIV-positive women of age 30-50. The wider age range for HIV-positive women was initially implemented because of the increased risk of precancerous cervical lesions and a more rapid progression to cancer among HIV-infected women. However, during the implementation phase, the risk of unintended disclosure of HIV status was observed and it was agreed to expand screening to the wider age range for all women. Figure 1 presents Rwanda's algorithm for cervical cancer screening and follow-up services. In accordance with this Screen, Notify, See, and Treat model of care delivery, all women are screened for HPV using the Qiagen *care*HPV test technology. This particular HPV test was chosen because of the opportunity of the MoH to partner with Qiagen. Furthermore, *care*HPV is cost effective, easy to use in remote settings, does not require complex laboratory equipment, and tests for 14 HR HPV types.^15–20^ Those who test HPV-positive undergo VIA to assess for the presence of cervical lesions, which, if present, are treated via cryotherapy or biopsied for further evaluation. Women with biopsies indicative of cervical cancer that cannot be treated by cryotherapy (large precancerous lesions or lesions in the endocervical canal) are referred for surgical management, either by loop electrosurgical excision procedure or complete hysterectomy. Women who test HPV-negative do not undergo VIA but are rescreened for HPV after 7 years if they are HIV-negative, or 3 years if HIV-positive. Women who are HPV-positive but VIA-negative are rescreened in 1 year, regardless of HPV genotype. The slow development of precancerous lesions into invasive cervical cancer allows for this extended screening interval.^21^ Village level recruitment by CHWs for screening activities ensures that patients return at the necessary intervals for future screening and/or follow-up visits.

In May 2013, training curricula for the Screen, Notify, See, and Treat model were delivered to health professionals at five district hospitals and 15 health centers, which were selected on the basis of service delivery capacity, regional representation, and availability of comprehensive cancer care. One medical doctor per district hospital, two nurses per health center, and nine laboratory technicians were trained for their respective roles in the screening program. Beginning in June 2013, standard meetings in Rwanda's 15,000 villages informed community health workers of the dates when a mobile team is planned to visit the health center for screening.^8^ Concurrently, social mobilization strategies including local radio broadcasts, church networks, and engagement with women's groups were used to raise cervical cancer awareness and educate women about the upcoming availability of screening services. Community health workers enrolled women for free screening and nurses then reported to the district level on how many reagents were needed and the length of time needed for each screening day. Upon arriving for her screening, every woman was informed on how samples will be collected. Each woman was asked to stay at the health center for a few hours to wait for her HPV test result.^8^ Beginning in September 2013, cervical cancer screening services became available at Muhima, Rwinkwavu, Gahini, Butaro and Ruhengeri District Hospitals, as well as 15 health centers within the hospitals’ catchment areas. Altogether, this geographic rollout brought coverage to a population of more than 1.3 million people.

## RESULTS

Between September 2013 and October 2014, a total of 7,520 women were tested through the screening program (Table 1). The percentages of the female population that received screening services during this time were 5.89%, 10.90%, 9.44%, 10.02%, and 1.01% in Muhima, Rwinkwavu, Gahini, Butaro, and Ruhengeri district hospitals, respectively. Of the 7,520 women tested, 874 (11.6%) women tested HPV-positive and 6,646 (88.4%) were HPV-negative.

**TABLE 1:**
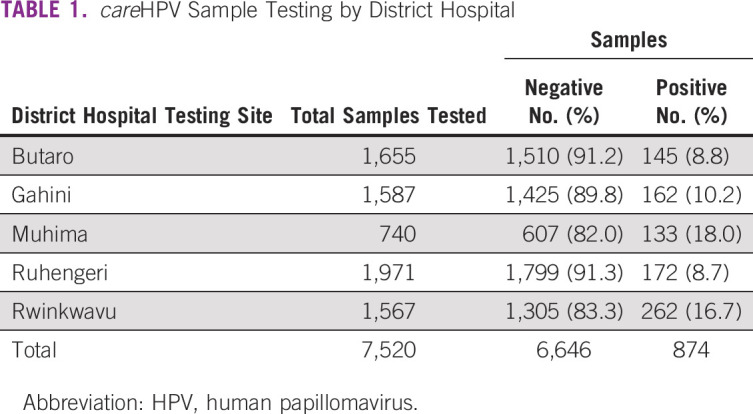
*care*HPV Sample Testing by District Hospital

Of those 874 who screened positive, 780 (89%) received VIA. The remaining 94 (11%) were lost to follow-up and did not receive VIA. This includes 11 women from Muhima hospital who provided nonfunctional contact details and 83 women who were waiting for VIA after the data collection period because of scheduling conflicts. Of the 780 women who received VIA, 204 (26%) were VIA-positive, 561 (72%) were VIA-negative, and 15 (1.9%) had suspected cancer and were referred for biopsy. Eight of those 15 women eventually underwent hysterectomy for treatment of advanced cervical cancer. Of the 204 women who were VIA-positive, 151 received cryotherapy. In accordance with the screening protocol, cryotherapy was administered on the same day as VIA for the majority of patients. The remaining 53 cases had to be deferred to later dates because of providers' lack of comfort with the procedure or shortage of cryotherapy gas. Notably, no patients underwent loop electrosurgical excision in the first year of the program.

Overall, the *care*HPV test technology met the needs of this screening program well, although several significant challenges did arise. At times, inadequate infrastructure led to power outages at Butaro, Gahini, and Rwinkwavu district hospitals. This caused approximately 20% of the *care*HPV test runs to fail, leading to waste of reagents and consumables. One of the 29 *care*HPV test systems used during the initial 12 months of screening ceased functioning and had to be returned to the manufacturer, reducing the efficiency of screening at the Ruhengeri health center.

Delays in service provision and loss to follow-up were caused by several factors. The 96-sample testing capacity of the *care*HPV system (including six controls) occasionally prevented same-day VIA and cryotherapy. Because the collection of 90 samples in a single morning was not feasible, some HPV-positive women were required to return to the health facility for VIA and cryotherapy during a second visit. Additional delays in the follow-up of HPV-positive women for VIA occurred because of low availability of trained providers. These barriers to providing same-day follow-up were exacerbated by hesitancy among eligible women to take advantage of screening services. To remedy this, health facilities normalized the process by establishing regular testing days at each center; this encouraged hesitant patients to present to the health facilities for screening. After the experience and testimony of those who were tested, women came in mass requesting to be screened. Loss to follow-up of HPV-positive women also occurred in the Kigali area when women were not known or referred by community health workers and contact information proved invalid.

## DISCUSSION

To our knowledge, the data described here represent the largest cohort to date of women screened for HPV through DNA testing in an African country. The first year of Rwanda's National Cervical Cancer Screening and Treatment Program succeeded in serving more than 7,000 women and referring more than 1,000 for further assessment and care. Challenges included patient loss to follow-up, lack of equipment and trained staff, and confidentiality regarding HIV status. Our findings corroborate existing literature in demonstrating that cervical cancer screening using HPV DNA testing is both effective and feasible in low- and middle-income settings.^22–24^ This is of particular importance given that the global disease burden of cervical cancer, a largely preventable disease, acts along socioeconomic lines, with nine of 10 deaths occurring in poor countries.^25^ Global elimination of cervical cancer is in sight, and Rwanda is leading the way in primary prevention through HPV vaccination. However, comprehensive cervical cancer programs that will make elimination a tangible reality will also include screening, treatment, and palliation. The WHO Global Strategy toward eliminating cervical cancer as a public health problem recommends that countries transition to HPV testing as the primary screening method to reach this goal.^26^ This study demonstrates the potential for success of expanded HPV screening in Rwanda and other low-resource settings.

During the first 12 months of Rwanda's cervical cancer screening rollout across five districts, many precancerous lesions were detected and treated and eight malignancies were addressed. There is no doubt that many lives were saved or extended by this program. This initial phase of screening could not continue, however, because of disruptions in funding with the MoH's partnership with Qiagen, ultimately leading to a cancellation of the partnership after the initial phase detailed here came to an end. The expected program rollout did not occur, and the expansion of *care*HPV screening services is currently on hold. Currently, screening for cervical cancer is done with Abbott molecular HPV tests at five district hospitals (one per province). Other health facilities are using VIA as a primary screening method, despite its lower sensitivity and specificity. The clear need and health benefit that could be brought by reinstating this comprehensive HPV screening program remain.

Prophylactic HPV vaccination is an effective cervical cancer primary prevention strategy that has been implemented with great success in Rwanda beginning in 2011. However, the resulting reduction in cancer incidence will not be appreciated for nearly 20 years,^27^ and the Gardasil vaccine protects against only four (HPV 6, 11, 16, and 18) of more than 100 known types of HPV.^28^ Because of these limitations, vaccination campaigns must be coupled with expanded screening and clinical services that accommodate all patients with cervical cancer, regardless of the strain of their HPV infection or their stage at diagnosis.

Rwanda's screening program with *care*HPV represents the first time that healthcare providers have screened for cervical cancer using a test that detects HPV DNA. Several longitudinal studies have demonstrated that laboratory testing for HPV DNA is more sensitive than both VIA and cytology and is more effective in reducing CIN2 lesions and cervical cancer mortality.^11,29,30^ It also predicts a longer period of low risk of developing precancerous lesions, allowing for screening intervals to be lengthened, an important programmatic element in developing countries where many women face barriers to accessing health centers. For these reasons, laboratory HPV testing is also one of the most cost effective methods in resource-limited settings.^18,31,32^ *Care*HPV test technology in particular is practical for low-income settings as it does not require air conditioning or running water and can be conducted by those with very little lab experience.^16^ A limitation of the *care*HPV test exemplified in this study is the need for a batch of 96 samples (including six controls) for optimal management of resources. Since collection of at least 90 samples in a single day was not always possible, some women were required to return to the facility to receive their result. Currently, technical innovations allowing for point-of-care HPV testing exist but remain too costly for widespread use in LMICs.^33,34^ However, the current WHO Global Strategy for cervical cancer elimination stresses the importance of market incentives to improve access to high performance diagnostics such as HPV tests.^34^

Several considerable challenges arose during the implementation period. One issue that became immediately apparent was the different age criteria for screening HIV-negative and HIV-positive patients, which had the potential to compromise patient privacy by inadvertently revealing HIV status. These confidentiality concerns were addressed through the revision of the age minimum for screening services to 30 years for all women regardless of HIV status, thereby ensuring that women with HIV receive the early screening they require without jeopardizing the confidentiality of their health history.

Additionally, a number of HPV-positive patients were lost to follow-up because of the collection of inaccurate or incomplete contact information, thereby preventing VIA and other follow-up services from reaching these women. Furthermore, as described above, same-day VIA was not always possible, and the number of healthcare professionals trained in VIA was limited; these two factors combined to create scheduling delays for follow-up appointments. Even in cases in which same-day VIA was provided, cryotherapy was not always administered immediately following VIA because of a lack of necessary resources or provider hesitancy. Once the high-coverage, low-volume model of care was determined to be ineffective for patient recruitment and service delivery, an alternative focused coverage, high-volume model was adopted. Under this new model, screening teams rotated among health facilities and CHWs arranged for women from their respective villages to attend screening services on predetermined days, thus increasing the efficiency of service delivery at each location and reducing delays in care. Standard practices were also amended to include verifying patient contact information at the time of the first screening visit to ensure that patients could be contacted for follow-up appointments if necessary.

In addition to patient loss to follow-up, variable methods of record keeping resulted in inconsistencies across the paper records maintained during the handover of samples between testing and laboratory facilities. To ensure that all samples were accounted for on both ends of these transactions, a standard procedure for real-time count verification during sample handover was implemented. Centrally driven quality control oversight was established to ensure compliance, adding a second layer of assurance. This mechanism of regulatory oversight both empowered district hospitals by granting ownership of their quality control practices and establishing a standard of regulation through documented consequences for inadequate performance.

Concerns regarding the efficient use and sustainable acquisition of consumables posed a significant financial and logistical challenge for the program. Assistance from the Centers for Disease Control and Prevention was required to procure a sufficient quantity of supplies within shelf life requirements. Once acquired, efforts to reduce unnecessary waste occasionally led to delayed care for patients. As no patients underwent loop electrosurgical excision procedure in the first year of the program, future cost considerations must include the cost-efficiency of maintaining this capacity at district hospitals.

In conclusion, the 12-month feasibility study conducted from September 2013 to October 2014 demonstrates that cervical cancer screening services using HPV testing provided through public health facilities in Rwanda can be successfully implemented and maintained in resource-limited settings. Despite initial challenges—including care provider training, patient hesitancy, loss to follow-up, and financial and logistic challenges—the program was executed successfully. In its first year of operation, screening services were made available to thousands of women who would not otherwise have received such care. Although future studies will be required to determine the population health impact and cost efficiency of the program, the success observed in this HPV screening program is promising of Rwanda's ability to develop a robust, well-informed cervical cancer screening program. Wider implementation and scale-up of HPV testing throughout Rwanda are recommended to reach the goal of cervical cancer elimination.
